# Experiences and Challenges of an English as a Medium of Instruction Course in Taiwan during COVID-19

**DOI:** 10.3390/ijerph182412920

**Published:** 2021-12-08

**Authors:** Shih-Ling Lin, Tzu-Hsing Wen, Gregory S. Ching, Yu-Chen Huang

**Affiliations:** 1 Graduate Institute of Educational Leadership and Development, Fu Jen Catholic University, New Taipei City 24205, Taiwan; happyangel040@gmail.com; 2 Office of Teacher Education and Careers Services, National Taichung University of Education, Taichung City 403454, Taiwan; kksunub@gmail.com; 3 Research and Development Center for Physical Education, Health, and Information Technology, Fu Jen Catholic University, New Taipei City 24205, Taiwan; 4 Bachelor’s Program in Educational Leadership and Technology Development, Fu Jen Catholic University, New Taipei City 24205, Taiwan; piperginny@gmail.com

**Keywords:** Taiwan, blended learning, student-centered, pedagogical design, English as a medium of instruction

## Abstract

Recently, Taiwan’s higher education has been impacted by COVID-19 and the necessity of English as a Medium of Instruction (EMI). In 2018, the Taiwanese government approved a roadmap for the development of a bilingual nation by 2030. This resulted in a renewed focus on EMI. However, the fluctuating surges of COVID-19 have caused university classes to shift from face-to-face to online. To assess its effectiveness, the current paper describes the quantitative and qualitative experiences and challenges associated with a blended EMI course within a private Taiwanese university. The data was collected from the students in the spring semester of 2020 (40 students) and 2021 (23 students). Overall satisfaction rate is calculated at 4.13; indicating that the transition from face-to-face to online has not affected the students’ overall satisfaction with the course. In addition, interviews and focus groups respondents pointed out the importance of a student-centered course approach and the opportunity to practice English in order to improve their competitiveness. While the flexibility offered by the blended learning approach during COVID-19 has given students more freedom to learn at their own pace. Lastly, in times of uncertainty, a careful pedagogical design will help to make the learning process fruitful and sustainable.

## 1. Introduction

Universities around the world face significant challenges and opportunities in the wake of the COVID-19 pandemic [1,2]. As a result of this, many countries have decided to close their schools in order to stop the spread of the disease, while implementing online learning systems as well [3,4,5]. Initially, schools in Taiwan continued to operate normally, and students had to attend classes as scheduled; however, strict regulations were put in place to ensure the safety of students and teachers [6]. Nonetheless, university faculties have begun integrating online instruction into face-to-face classes following COVID-19′s sporadic spread in 2020 [7,8], while shifting to full online learning from May 2021 continuing until the end of the semester [9,10]. For this reason, assessing the experiences and challenges of both teachers and students is equally important to determine the effects of these shifting modalities of learning [11,12,13].

Recently, promoting English language proficiency is another important component of Taiwan’s higher education. With the aim of raising the English proficiency of the public and improving Taiwan’s overall competitiveness by 2030, the government’s executive branch passed a blueprint for the development of a bilingual nation in 2018 [14]. Consequently, the Taiwanese government enacts a variety of legislative reforms and proposals aimed at improving the ability of all individuals to communicate in English [15]. To support the national goal, more courses are being offered using the paradigm of English as a medium of instruction (EMI), not only to attract international students, but also to provide Taiwanese students with the chance to enhance their competitiveness through global mobility opportunities [16]. As a result of these goals, many higher education institutions in Taiwan are re-evaluating the possibility of offering full English taught degree programs, though this may vary greatly depending on the discipline or field of study [17]. For example, full English programs in the medical sciences (e.g., medicine, nursing, biology, etc.) and engineering sciences (chemistry, physics, etc.) might be acceptable, while humanities and social sciences will be less attractive and likely limited to a few local students. As a complement to these full-time degree programs in English, stand-alone EMI course subjects are gaining popularity [18].

EMI is not just a Taiwan phenomenon or a trend in countries that do not speak English. A large number of countries have adapted EMI courses to their curriculums as a result of globalization and internationalization [18,19,20]. Moreover, higher education institutions are embracing a holistic approach to internationalization as part of the Education for Sustainable Development (ESD) goals of the United Nations Education, Scientific and Cultural Organization (UNESCO) [21].

Importantly, the situation of COVID-19 in 2020 has led to a sudden shift from traditional face-to-face to online instruction, which was initially met with reluctance from many faculties [22]. This shift to online became compulsory with the resurgence of COVID-19 in May 2021, however, this also gave rise to problems of social and economic inequalities [23]. In reality, the move to online instruction has impacted many courses that are typically offered in traditional face-to-face teaching [24]. For online courses during COVID-19, student-faculty interaction is deemed a reliable indicator of student learning satisfaction [25,26]. This also applies to EMI courses, where actual interaction is also seen as a determining factor in course satisfaction [27]. However, this may not always be the case, as previous online learning experiences are considered a major proponent of improving performance than interaction [28].

In Taiwan, most teachers receive extra credit hours or some kind of financial incentive to teach EMI courses [18]. To understand their effectiveness, universities have required the submission of course outlines, sample lectures, and course reflections at the end of each semester. However, for a truly effective EMI teaching and learning process, further analysis is still needed [20,29]. Importantly, with the recent shift from face-to-face to online instruction, it is also necessary to gain a deeper understanding of the experiences and challenges associated with the blended EMI courses. Accordingly, this paper attempts to answer the following research questions:

- What are the course attitudes and satisfaction of the students?
- What distinct experiences and challenges did the students and instructor face during the EMI course?

## 2. EMI in Taiwan

EMI is very similar to bilingual education. In the teaching-learning process, bilingual education is defined as the teaching of academic content in two ways: using the native language (or first language) and in a second language (which is usually English) [30,31]. Bilingual education is actually associated with both positive and negative outcomes [32,33]. For instance, due to a limited number of classroom contact hours, bilingual students will tend to know fewer vocabulary families, resulting in less satisfactory verbal recall performance [33]. Furthermore, learning in the student’s first language remains the preferred method, as it will most likely bridge the gap between home and school cultures [32]. In contrast, a seminal study of 12 bilingual programs found that bilingual students’ English language achievement was higher in some instances than the national average [34]. Additionally, an examination of Guatemalan bilingual schools revealed that students who attend bilingual schools have higher attendance and promotion rates, as well as achieving higher academic scores in all subject areas [35]. Similarly, a review of nearly two decades of research and practice in bilingual teaching within Chinese higher education demonstrated the overall effectiveness of such an educational design [31].

In practice, many countries such as China have tried to use bilingual English-Chinese courses to enhance students’ foreign language competencies [36]. Almost always, bilingual education is also practiced formally and/or informally within Central Asia [37], East Asia [38], and Southeast Asian countries [39]. In Spain, besides teaching English as a foreign language, English is also used in teaching Science, History, and Geography [40]. This pedagogy of using English to teach specific subjects is quite similar to the concepts of Content and Language Integrated Learning (CLIL); which refers to any learning method that involves the use of a second language or the simultaneous use of two languages to learn the lesson content [41]. In Taiwan, the concept of CLIL encompasses and overlaps with both bilingual education and EMI courses [18]. In practice, CLIL is about the use of a foreign language or a lingua franca, not a second language. CLIL also implies that teachers are typically non-native speakers of the target language, while the majority of CLIL courses use English. In addition, less than half of the curriculum is typically taught in the target language. Finally, CLIL is typically used when learners have already acquired reading and writing skills in their first language [42]. Despite minor conceptual differences, the idea of developing foreign language proficiency in students is still the same.

In Taiwan, a study was conducted to investigate university students’ performance after taking a CLIL course. Interestingly, results indicate that students’ self-perceived receptive and productive English competences still improved after they left school for nearly two years [43]. Furthermore, a review of 92 CLIL programs in Taiwan shows that students’ perceptions of CLIL courses are determined by their English language proficiency [44]. Simply put, the more proficient the students are in English, the more they will appreciate CLIL courses. In Taiwan, bilingual education policies are mostly established to facilitate international student enrolments [45]. Moreover, bilingual education in Taiwan might also include the combination of local languages and dialects such as Mandarin Chinese, Hakka (the language spoken by the Hakka people), and Southern Min (sometimes referred to as Minnan or Taiwanese Hokkien), instead of English [46]. Furthermore, for some faculties in Taiwan, allowing students to choose the medium of instruction is also an effective way to deal with linguistic diversity [47].

EMI programs in Taiwan are not new [18]. EMI courses are widely regarded as necessary in preparing Taiwanese students for the globalized world, however, the effectiveness of these courses is still contested [48]. Findings from a study of 370 university students and six professors from three different universities in Taiwan show that, despite not expressing any negative attitudes towards the EMI courses, students did not exhibit a high level of self-perceived English language comprehension [49]. Additionally, in a survey of 476 students enrolled in 25 EMI courses across six Taiwanese universities, findings revealed that students attributed their learning difficulties to their own level of English language proficiency [50]. In other words, it appears that the effectiveness of EMI courses is still very much determined by the students’ level of English proficiency.

According to research, there are several perceived advantages to EMI, including the increase of students’ confidence in their ability to understand and speak English [51], at the same time, it enhances the tendency to engage with others [52,53]. Furthermore, motivated students in EMI courses are more likely to engage in their learning and make greater gains in cognitive understanding [29,54]. Apart from the perceived high level of student satisfaction with EMI [55,56], there are also some negative issues of EMI. For instance, the uneven level of English proficiency within the class may cause some students to feel excluded and even led to believe that they are less capable than their peers [57]. Moreover, the implementation of EMI courses has also been hindered by several challenges, such as the teachers’ lack of proficiency in the English language, low parental involvement, the fear of losing one’s own culture and mother tongue, and the lack of funding from the government [58]. Importantly, several important issues should be considered when designing an EMI course, such as the balance between lesson content and language, the relative importance of learning the English language for students from different cultures, and the inequalities regarding assessment when using another language [59].

Some Taiwanese studies have noted that teachers are the key to students’ course selection (or enrollment) [50,60]. This means students will enroll in specific EMI courses according to who teaches them. Therefore, how teachers teach EMI courses still has a great deal of significance. Researchers have also noted that it is difficult to measure the quality and effectiveness of an EMI program [61]. Others have noted that the quality of interactions matters, as evidenced by whether or not students ask questions and take part in the class discussion [49]. In addition, a faculty that is able to apply code-switching within the pedagogy; using the students’ local (or native) language for a moment before switching back to English, will tend to enhance the overall EMI learning experience [60].

In Taiwan, local Taiwanese faculty are sometimes hesitant in teaching EMI courses. Some have mentioned that local teachers have been expressing concerns over the practicality of enacting EMIs, while criticizing institutional policies at the same time [62]. Despite the apparent value of the EMI courses, there is a lack of practical applications of the English language in Taiwan, which is causing many teachers to doubt their actual usefulness [63].

## 3. Blended Learning during COVID-19

Blended learning or hybrid teaching describes the process of mixing face-to-face instruction with computer-based teaching [64,65]. With the emergence of new technologies, blended learning concepts are constantly being expanded and developed [66]. In today’s educational environment, technology is widespread in all aspects of the teaching and learning process and is therefore considered to be blended in a broader sense [67]. More importantly, with COVID-19 making face-to-face instruction difficult, blended or hybrid learning seems like the ideal solution [68,69].

In an attempt to better understand blended learning, many have tried to categorize and organize its concepts. Staker and Horn [70] proposed four distinct models: Rotational model in which students alternate between face-to-face and online learning. Flex model in which students use a combination of online and face-to-face learning methods, with the teacher participating in face-to-face support sessions. Self-blending model in which a student can take an online version of a classroom course as a supplement. Enriched virtual model where students can participate in both face-to-face and online classes. The most popular approach, however, is to categorize blended learning in terms of space, time, fidelity, and humanness [64]. Space refers to live teaching (physical or face to face) or virtual (distributed), time refers to live synchronous (very short delay time) or asynchronous (long delay time), fidelity refers to high (rich in all senses) or low (text only), and humanness refers to high human (no machine) or no human (high machine).

Amid the current COVID-19 pandemic, blended learning designs are widespread. In France, video conferencing was used to teach medical students [71], while in the United States, virtual learning was used to teach surgical training [72]. Some even conduct physical education classes using a combination of online instructions and asynchronous activities [73]. This type of educational design is also seen in engineering students, wherein a combination of online classes and pre-recorded videos enhances the overall learning experience during COVID-19 [74]. This proves that asynchronous videos (pre-recorded) can also be used to effectively fuel engagement [75]. However, a disadvantage of the blended approach is the reduction in social interaction (student-faculty, student-student), which is key to fostering engagement and participation [76]. Nonetheless, blended learning can also help students with unstable internet connections, for whom the quality of live synchronous learning is difficult to achieve [77,78].

## 4. Materials and Methods

### 4.1. Research Design and Participants

The current research is structured as a case study, wherein an in-depth inquiry of a contemporary phenomenon is conducted in its real-life context [79,80]. For this particular study, the focus is on the blended EMI course “Globalization and Higher Education” within a comprehensive university situated in the Northern area of Taiwan. Additionally, the current study is framed as a mixed-method research, in which paradigms from both qualitative and quantitative approaches are applied during the data collection [81]. Qualitative data were collected from instructor reflection logs, semi-structured interviews with randomly selected students, and focus groups conducted at the end of the semester. Individual semi-structured interviews are used to gather the students’ experiences and challenges that they encountered during the duration of the blended EMI course. While, focus group interviews consist of five to six students per group and tasked with describing their perceived objectives, strength, weakness, opportunity, and threats with regards to the EMI courses offered by the university. In addition, quantitative data were collected using a course satisfaction and learning perception survey administered by the university two weeks before the end of the semester.

Data for the current study were collected from students who were enrolled in the spring semesters blended EMI course “Globalization and Higher Education” during 2020 and 2021. Participants of the study consisted of 63 students (40 from 2020 and 23 from 2021). Online instructions were delivered through Microsoft Teams and the university’s learning management system. Since this is an elective course, students are drawn from a variety of colleges and year levels. For the 2020 spring semester, a total of 25 female and 15 male students took part in the course. Of the 40 students, 14 are freshmen, 11 are second-year students, 5 are juniors, and 10 are senior graduating students. The different course majors are foreign languages (9 students), engineering and natural sciences (9), business and management related courses (6), social sciences (5), arts and religious sciences (5), medical sciences (3), and law (3). As for the 2021 spring semester, a total of 14 female and 9 male students took part in the course. Of the 23 students, 4 are freshmen, 10 are second-year students, 6 are juniors, and 3 are senior graduating students. The different course majors are business and management related courses (6 students), foreign languages (4), medical sciences (4), law (4), arts and religious sciences (3), engineering and natural sciences (1), and social sciences (1). Lastly, average final grades for the 2020 class are calculated at 83.07 and 89.45 for the 2021 class.

Regarding ethical considerations, the current study was carried out in accordance with the Declaration of Helsinki. Students were informed at the beginning of the semester of the purpose of the study and to what type of data will be collected and analyzed. Consent was obtained from all of the students. Furthermore, data for this study was collected within an accepted educational setting and as part of the normative educational practices with opportunities for learning from the findings. In addition, the interview participants were given pseudonyms based on a coding scheme [82,83]. Lastly, identifiers for the student participants are masked by the information obtained in such a way that, directly or indirectly, they cannot be easily determined.

### 4.2. The EMI Course

“Globalization and Higher Education” is an engaging elective course. Through this course, students will be able to identify the key concepts that shape their current understanding of globalization and its impact on education around the world. In order to better understand the multi-faceted phenomenon of globalization, discussions will also include, but will not be limited to, theories from the fields of comparative and international education, philosophy, sociology, economics, political science, and anthropology.

The following points are highlighted within the course:

- To learn about the history of globalization and to explain the arguments against and for globalization;
- To become familiar with additional concepts and issues that are parallel to globalization, such as internationalization, study abroad, university league tables, and so on;
- To identify and assess how globalization affects the major activities in our daily lives;
- To analyze and identify the major issues surrounding globalization and education;
- To analyze the governance of schools and the changes that take place within Taiwan and elsewhere; and
- Lastly, to enhance the ability to communicate oral and written ideas using English within a classroom setting.

The entire duration of the course is 18 weeks. Weeks 1 to 6 are dedicated for lecture sessions, wherein the instructor provides assigned readings and in-class discussions with the use of PowerPoint presentations. Topics for weeks 1 to 6 include the definition of globalization and the various issues surrounding globalization. In class activities for class motivation and lesson interactions, includes group dynamic activities, ice breakers, concept mapping (mind mapping), individual sharing, group discussions with guide questions, and role playing. Week 7 is to watch a movie about globalization and provide some insights and reflections based on the guide questions. Week 8 is for the individual consultation of the students with the instructor regarding their final individual oral report topics. Week 9 is research day for the students in preparation for their final individual reports. Weeks 10 to 17 are for the individual reporting. Scheduling for the individual reports is accomplished through a lottery. Reporters are also required to upload their presentation to the class’s learning management system a day before their scheduled report. During the report, the student audience is required to write some comments, questions, and or report highlights, which are collected after the class. A discussion of the reports led by the instructor takes place after all the students scheduled for the day have reported. Week 18 is the deadline for the submission of written reports; at the same time, focus group interviews are also conducted. Moreover, semi-structured interviews were also randomly accomplished after the class starting from weeks 7 to 17.

Blended learning sessions include watching a film about globalization (asynchronous) and individual discussions (synchronous) during the 2020 semester, while all individual reporting is done live (or synchronous) using Microsoft teams, with follow-up activities accomplished at the students’ own pace.

### 4.3. Data Analysis

Quantitative data from the course satisfaction and learning perception survey were analyzed by computing for the mean (or average) and percentage of the items. For the individual semi-structured interviews, students’ experiences and perceived challenges were analyzed, categorized, and repeating themes noted together with the instructor’s reflection logs [84]. Furthermore, Voyant Tools was used to analyze the focus-group responses for trends and relationships [85]. Visualization of the data was intentionally chosen to support better understanding of interpretations [86,87]. For instance, the most common words used by the students were displayed as keyword cloud, in decreasing order of frequency (indicated by large to small font size) [88]. Moreover, bubblelines and word trends were included to visualize the frequency and distribution of the five most common keywords [89]. Lastly, close proximity links between the five keywords are shown to provide an understanding of how the terms are related [90,91].

## 5. Results

The following section sums up the experiences and challenges encountered within the blended EMI course on “Globalization and Higher Education”. In the first section, quantitative data collected from the course satisfaction and learning perception survey are reported. Information presented included the students’ class attendance and attitudes, as well as their perception of course satisfaction. The second section contains a synthesis of the qualitative information gathered from the focus groups and semi-structured interviews, and instructor reflections.

### 5.1. Students’ Course Attitudes and Satisfaction

In the current study, quantitative data is collected by using the schools’ system-generated course satisfaction survey. A few weeks before the semester ends, the school will automatically send an email to every student informing them of the need to complete the course satisfaction survey. It is not mandatory for the students to complete the survey, so it is up to them whether they choose to complete it or not. Table 1 and Table 2 provide the information on course attendance, class attitudes, and course satisfaction based on self-reported students’ responses. The results from the survey indicated that most students attend class most days, and that most have only one or two absences. It should be noted that a majority of the additional absences during the spring 2020 semester were attributed to sporadic COVID-19. Studies show that the reasons students skip classes at universities are quite difficult to categorize, however, elective courses should have better attendance rates [92].

Besides the attendance, additional classroom attitudes were also asked. Students are asked to reply either yes or no for the five statements (see Table 1, Students’ attitude). Results show that less than half of the class comes to school prepared (43% for 2020 and 33% for 2021); however, most of the students would listen attentively (85% for 2020 and 67% for 2021) and participate during class activities (93% for 2020 and 75% for 2021). Furthermore, more than half of the students accomplished their assigned tasks on time, such as during the individual final reporting and uploading of their presentation (68% for 2020 and 75% for 2021). Upon further clarification, the need for re-scheduling of their final reports is mostly due to unforeseen conflicts with their other classes. For the 2020 class, additional re-scheduling was needed to adjust for the COVID-19 situation.

For the students’ satisfaction with the course, Table 2 showed the results of the 10 statements asked regarding their perceived gains and course comments. Students are asked to rate their perceived gains and satisfaction using a 5-point Likert [93] type scale with 1 as the least and 5 as the most. Overall course satisfaction was computed at 4.13, denoting moderately high satisfaction. Furthermore, students rated almost all of the statements with moderate to moderately high satisfactions and gains. This is actually quite promising, wherein students are able to acknowledged whether they are able to learn something from the course. Further clarifications are provided in the following qualitative data analysis section.

### 5.2. EMI Experiences and Challenges—Individual Interviews

Presented here are the results of semi-structured interviews with the students to determine how they perceived the course, which are analyzed together with the instructor’s own reflections. Data are analyzed, categorized, and typical themes were identified and discussed [84]. In total, 18 individual semi-structured interviews (11 from 2020 and 7 from 2021) were accomplished.

In terms of students’ experiences, interviews revealed that most students value the group dynamics (team building) and icebreaker sessions that precede the lessons.

*I was quite motivated with the first day of class, wherein the teacher explained the importance of listening to others with the use of the blind-folded paper activity.*

(2020 B03)

*For me it was the tower building activity that I like… I think the teacher’s objective is to tell us the importance of teamwork and collaboration.*

(2021 G07)

**Motivating students for collaborative learning** is a very important aspect of teaching nowadays. In order to foster learning with one another, motivation for effective group work or collaborative learning is considered an essential component [94,95]. Moreover, because the majority of class activities within the EMI course involved students working together, listening and collaboration should be stressed at the beginning of the semester.

*I believe that students learn better while discussing with their classmates… Although using English is not mandatory during their group discussions, but students still have to present their answers in English. Practice makes perfect.*

(Teacher 202004)

Furthermore, students are provided with many opportunities to use English during class. In order to learn a foreign language, it is important to have the opportunity to practice [96,97]. Within an EMI course, this can create a dilemma for the instructor. The issue of either **focusing on the lesson content or the teaching of the English language** is one of the important challenges for the teachers of EMI [98].

*After teaching this course for the second time, while letting the students use English during class, many students would ask for assistance with their grammars, vocabulary usage, and many other language related issues. This is taking too much of my class time.*

(Teacher 202105)

*It is really wonderful that I get the chance to use English during class… but sometimes I don’t know what vocabulary to use.*

(2021 B2)

*I get distracted easily… I have to translate using my smartphone before I am able to understand the lesson.*

(2020 B3)

Additionally, the **use of technology** enhances the learning process. Students are allowed to use a digital dictionary or smartphone in class (sometimes students will also bring their laptops or tablets) to translate unfamiliar words. However, too much reliance on technology could cause some problems, such as plagiarism of their reports (cut and paste from the internet) [99].

*As I told my class all the time, do not just cut and paste from the internet. Besides I think the report topic that you choose should be interesting for you, so there should be no problem.*

(Teacher 202006)

Students’ final individual reports are based on topics developed after discussion with the instructor. The design of the course is deliberately **student-centered**. Writing activities that are learner-centered (or student-centered), in which students choose their own topic, tend to engage students more [100]. For instance, students’ writing is actually highly influence by their cultural background [101], both the Malaysian and Japanese students choose to write about a comparative analysis between studying in their home country and Taiwan. Hence, almost all of the students choose a topic that is familiar to them.

Students are also required to submit a written essay or synthesis of the report in addition to the oral presentation. This is a conventional writing assignment that is often referred to as theme writing or school writing [102]. According to studies, writing activities are quite important for students to develop and enhance their writing skills [103]. However, there is the constant fear of students copying their work from the internet, which is highly undesirable [104]. Nonetheless, students are encouraged to use their own words and refrain from copying from the internet.

On the subject of shifting from face-to-face to online instruction, both students and instructor reported some difficulties with the initial transition in 2020. The majority of the problems are focused on class scheduling and clarification of the learning modes (online synchronous using Microsoft Teams or asynchronous with the use of the learning management system—fulfilling the assigned tasks).

*Not completely sure on the schedule of the classes… when I am supposed to go to school and when the classes are held online.*

(2020 G02)

*At first I am not sure whether I have to be online at the same time or I will just do the assigned tasks provided by the teacher.*

(2020 B3)

*Using the learning management system is suitable for asynchronous learning, while teams is for synchronous real-time learning… which require some time to get used to. I think students will have some difficulty at the beginning of the switch to online.*

(Teacher 202009)

In 2021, the students and instructor are already familiar with online instruction. As long as the scheduling is clear, the students can keep up with the lesson and the assigned tasks. In addition, students all noted the **flexibility** of the blended learning. Flexible in terms of students are able to complete the assigned readings and related tasks at their own pace. In other words, students are already quite accustomed to online learning.

*I think online learning is okay, I can stay at home and study… I have more time to accomplished the homework, it is very helpful and flexible.*

(2021 G01)

*Some lessons such as lectures are very suitable for asynchronous learning, while Microsoft Teams is more useful during the synchronous real-time individual reporting of the students.*

(Teacher 202104)

*Online learning is just the same for me, besides getting used to speaking in front of the computer, there is actually not much difference.*

(2021 B3)

### 5.3. EMI Experiences and Challenges—Focus Groups Interviews

**Figure 1:**
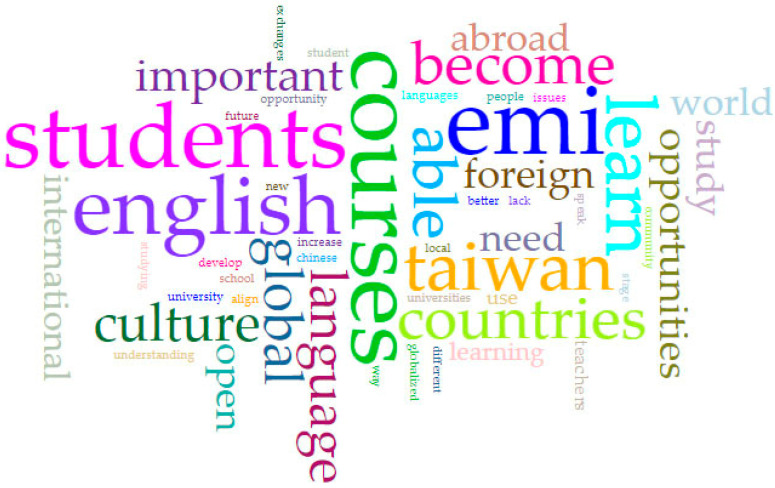
Keyword cloud for focus groups interview results.

For the focus group interviews, a total of 13 sessions were accomplished (eight from 2020 and five from 2021) with four to six students per group. Students were tasked with describing their perceived objectives, strength, weakness, opportunity, and threats with regards to the EMI courses offered by the university. Students’ responses were collected and analyzed together using Voyant tools. A total of 324 unique words were analyzed and organized together to form a keyword cloud (see Figure 1). Table 3 also contains the list of the top 20 keywords. By viewing the words from a graphical perspective, the most frequently used words can be easily determined (represented by decreasing font size; i.e., the larger the text, the more frequent the use of the word) [86,105].

Figure 1 shows that students recognize the importance of English as an international (or global) language as well as the need to learn a foreign culture either in Taiwan or abroad. Nevertheless, keywords are to be interpreted with caution since they do not reflect collocations, co-occurrences, or possible meaning variations [106]. For a more meaningful interpretation, a collocates graph or a graphical representation of the links between the high-frequency words is needed [91,107].

**Figure 2:**
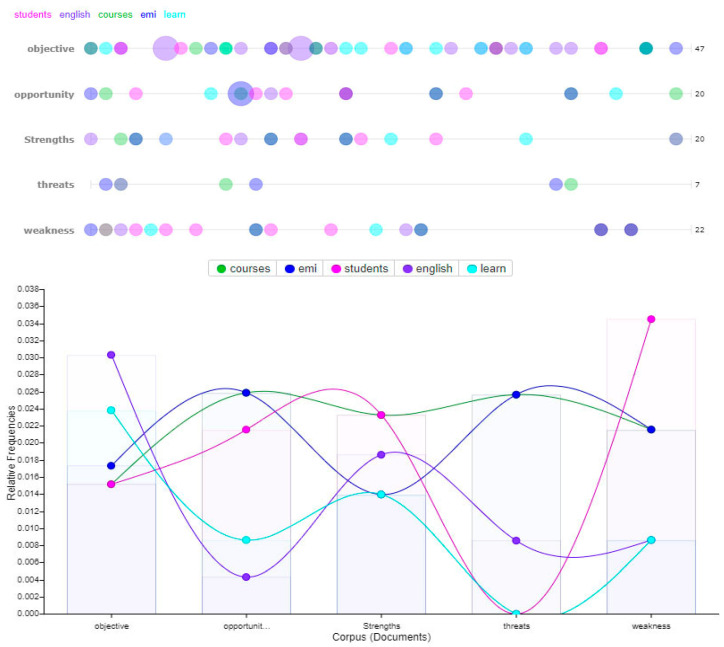
Graphical representation of the frequency of occurrence for the five main keywords.

A visual representation of the five main keywords was also provided in Figure 2, wherein bubblelines and word trends are used to help visualize their frequency and distribution [89,107]. Note that the various colors represent the different keywords. The highest ranking keywords are students, English, course, EMI, and learn. In total 116 instances were recorded. To help visualize, a trends graph is also provided for the five main keywords that represent the frequency of terms across the different responses (as indicated by objectives, strength, weakness, opportunity, and threats) [108].

**Figure 3:**
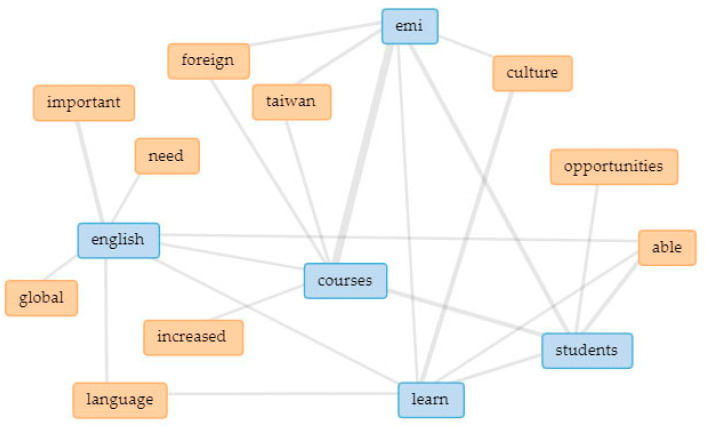
Interrelationships and extended links among the five main keywords.

**Figure 4:**
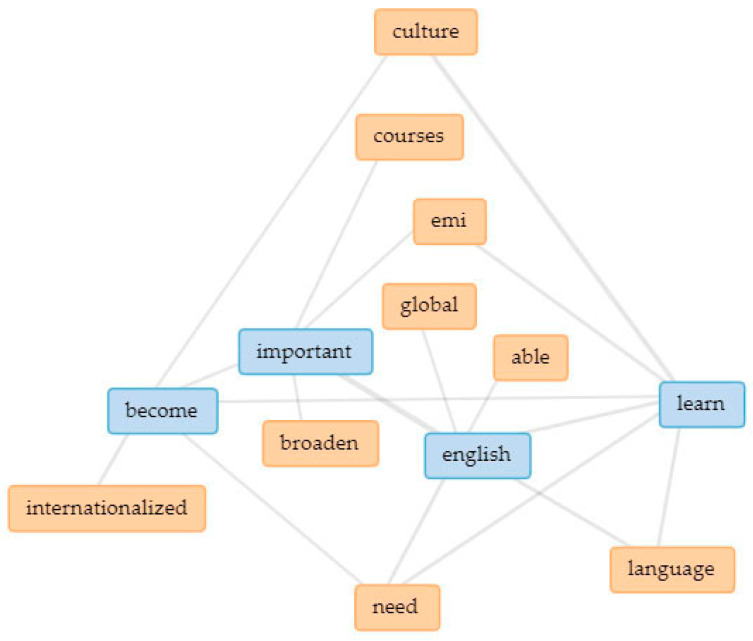
Interrelationships and extended links among the perceived EMI objectives.

**Figure 5:**
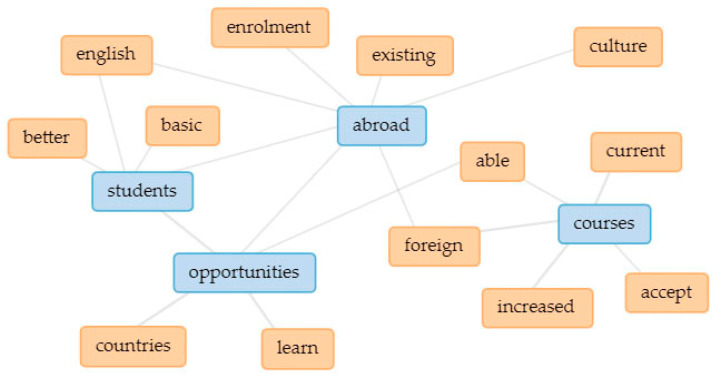
Interrelationships and extended links among the perceived EMI strengths.

**Figure 6:**
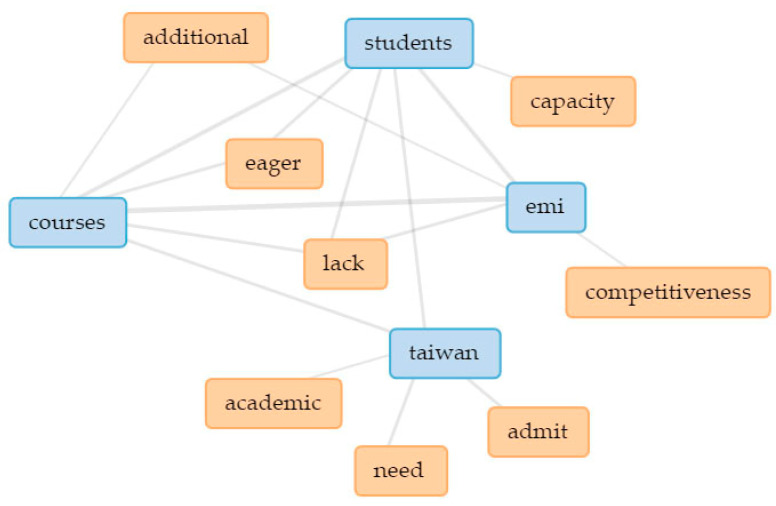
Interrelationships and extended links among the perceived EMI weakness.

**Figure 7:**
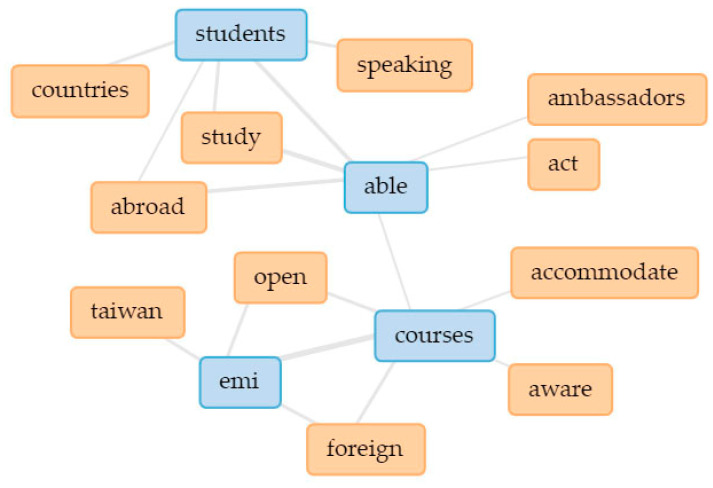
Interrelationships and extended links among the perceived EMI opportunities.

**Figure 8:**
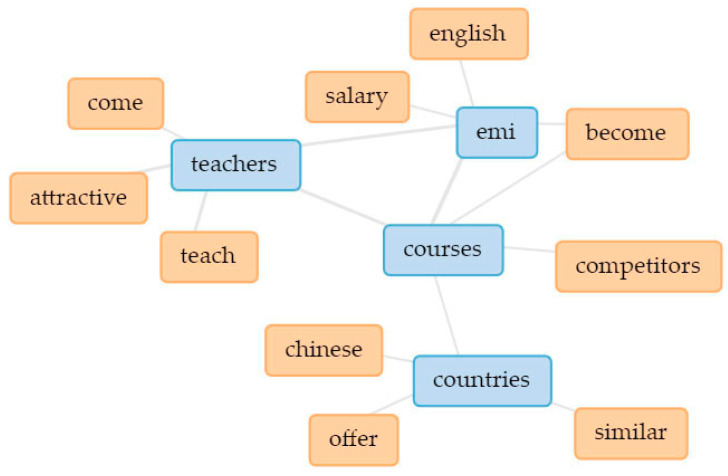
Interrelationships and extended links among the perceived EMI threats.

In Figure 3, the five main keywords are linked in a collocates graph. Linked keywords indicate how they are clustered together in the text (thicker lines signify higher occurrence), thus indicating underlying themes [109,110]. In addition, separate collocate graphs (Figure 4, Figure 5, Figure 6, Figure 7 and Figure 8) are also provided to illustrate the different themes within the perceived objectives, strengths, weaknesses, opportunities, and threats with regards to the EMI courses. Note also that blue text are the main keywords, while the orange ones are the common collocates.

For the overall collocates graph of the five main keywords, Figure 3 shows several emerging themes, which are quite similar to the earlier assumptions:

- Importance of English as a global language;
- Opportunities for students to learn a foreign language and culture; and
- Increasing trend of EMI courses in Taiwan.

In addition to English as a global language, another important aspect of EMI objectives emerged (see Figure 4). The importance of having a broad perspective on things (or to become more internationalized) is considered to be an important goal of the EMI program in Taiwan. EMI has long been regarded by Taiwanese higher education institutions as an **important strategy to internationalization** [111,112,113]. As well as improving English language proficiency among Taiwanese students, EMI courses also contribute to the holistic approach to internationalizing universities, one important aspect of which is to have a common language for communication [45].

In terms of perceived strengths, in addition to learning a foreign culture, EMI courses also tend to attract foreign students. Additionally, local students have an increased opportunity to participate in study abroad exchanges (see Figure 5). Moreover, they are also part of the strategies for internationalizing higher education [111,112].

In terms of perceived weaknesses, focus group interview results revealed a lack of both appropriate EMI courses and students who are keen to enroll in these courses (see Figure 6). There might be a number of reasons for the **mismatch between EMI course offerings and students**. Previous EMI studies suggested that there might be mismatches due to the students’ English language proficiency [114], the teacher’s teaching beliefs [115], and the course content itself [116].

With regard to the opportunities provided by EMI courses, students emphasized the importance of **being able to practice English** as well as being able to meet students from other countries (see Figure 7). EMI courses in Taiwan are usually combined with language training opportunities [18]; learning a foreign language through actual interactions and engagement is still considered an effective strategy [113].

Finally, the perceived threat of EMI courses is the increased hiring of foreign language teachers, which may result in the outflow of Taiwanese talent (see Figure 8). In fact, this phenomenon entails both brain gain and brain drain, where **talents are exchanged** [117,118].

## 6. Limitations

The primary objective of the current study is to determine and understand the various distinct experiences and challenges encountered by both the students and instructor of a blended EMI course in Taiwan. A mixed method research paradigm was used to collect both quantitative and qualitative data with a number of unique findings identified. The current study also has limitations. As the blended EMI course “Globalization and Higher Education” is mainly a social science course, results might differ from those of other medical sciences or STEM related courses. Furthermore, the current reported findings are based on one EMI course, where the results may not be representative of all the EMI courses offered by the university.

## 7. Discussions

With regards to the quantitative perspectives of course attitudes and satisfaction, the overall scores for the two semesters were computed as slightly high, which is typical for many elective EMI courses in Taiwan [119], regardless of being blended or not. This is actually due to the elective nature of the course. An elective course is one that students have the option to take as part of their program of study. It was found that more than half of the EMI courses in Taiwan are actually electives [50]. Importantly, since the students are the ones who decided to enroll in the course, they are most likely interested in the subject matter and therefore listen attentively and are engaged (or interact) more in class. Additionally, many EMI courses are also a kind of English for Specific Purposes (ESP), where the instructional content is confined to a particular set of vocabulary or subject area that is related to a certain profession [120]. Therefore, EMI courses that are directly related to a student’s future career should get better participation and satisfaction [121,122].

In terms of the qualitative perspectives, several distinct findings emerged regarding the experiences the students and instructor face during the EMI course. Within the course design, it is found that the best way to engage students in learning is to connect their interests with what they learn in school. In other words, a student-centered learning (SCL) approach [123]. The SCL design enables students to become motivated, simply put, as students slowly become responsible for their own learning, they gradually become self-directed learners [124]. For instance, students were asked to write and present an oral report on an educational topic that is both familiar and related to their current course in EMI. Additionally, through individual consultations with students, the traditional teacher-centered model gradually became a student-centered one [125].

Besides SCL, the EMI course is also designed to promote learning motivation through the creation of a collaborative learning environment, which leads to authentic learning processes. As a result, in-class activities enable students to relate what they learn at school to real-world issues, problems, and applications, thereby creating an authentic learning experience [126]. Furthermore, besides having ample opportunities to practice speaking English in class, the activities are also designed so that the students are required to work in groups. In fact, EMI courses encourage students to work in groups as a way to encourage more active participation [127]. By doing so, students are motivated to learn, and the classroom content is more engaging.

A blended approach to online instruction is also a form of SCL [128,129]. Although online learning is becoming more popular with the current generation of students [130], the blended approach is still the preferred model. Studies have noted that the flexibility provided by asynchronous and synchronous learning increases student satisfaction and efficiency [131]. Blended learning approach can also help students distinguish between learning tasks that can be completed at their own pace and those that should be done synchronously [132,133].

Findings also establish that Taiwanese universities are offering EMI courses as part of their internationalization efforts, which are in response to the globalization drive of academia [134]. The addition of EMI courses to most Taiwanese universities is actually one of the strategies to achieve sustainable internationalization [135]. Additionally, EMI courses can also help increase the number of international students enrolled at Taiwanese universities. Furthermore, besides having increased study abroad opportunities, local Taiwanese students also have the opportunity to learn in an environment similar to that in foreign institutions [113].

For the challenges of the EMI course, one perennial concern for the instructor is the balance between lesson content and teaching the English language. EMI courses sometimes experience this, where the attention is temporarily focused on language instead of content [136]. The situation is totally understandable, whereby the students would not be able to progress in the lesson if a language barrier prevented them from understanding the lesson [137]. Some practitioners of EMI have suggested the use of code-switching (temporarily using the local language to provide some keywords) to help facilitate the learning process [138,139]. Nonetheless, teachers should be careful on when and how the code-switching occurs [140,141,142].

Another concern is the prevalent use of technology in EMI courses. In some studies, technology does indeed increase a student’s vocabulary proficiency [143], but this may also be dependent on the student’s ability to use it well [144]. However, too much reliance on information technology can also result in plagiarism and cut-and-paste issues [145,146]. Furthermore, several issues of mismatch between EMI course offerings and students were also noted. Students’ hesitation to pursue EMI courses is related to an insufficient selection of suitable EMI courses for them and their inadequate level of English proficiency. Hence, the need for a more student-centered approach in overall EMI course design. Lastly, a perceived threat to EMI courses is the probable outflow of Taiwanese talent and inflow of foreign language teachers. As stated previously, this phenomenon implies both brain gain and brain drain; a sort of talent exchange or talent circulation [147], which is becoming common in EMI prevalent countries [148].

## 8. Conclusions

In sum, with the uncertainty surrounding the COVID-19 pandemic still lingering, blended learning offers an option that could enable flexible learning. Although schools in Taiwan have returned to face-to-face instructions, many schools have adapted the blended learning approach for greater flexibility. For instance, some classes are split into two groups, wherein each group alternately attend face-to-face classes, while the other group are using online (synchronous). In addition, as the need for improving Taiwanese students’ English language proficiency increases, it is also essential to develop a sustainable strategy. Using pragmatic student-centered strategies, content knowledge and language skills can be developed. Moreover, as Taiwan’s universities are also increasingly employing English in a variety of academic practices, in addition to gradually teaching many courses in English. This includes English-speaking administrative staff and a strong emphasis on English proficiency among new faculty recruits. Thus, as the importance of the English language grows, diverse neoliberal institutional management policies are likely to emerge. However, schools should still realize that no one policy fits all. There are no shortcuts, and change does not happen overnight. In the end, a sustainable EMI strategy no matter blended or not, still depends on careful pedagogical planning.

## Figures and Tables

**Table 1:** Students’ attendance and course attitudes.

Year (Spring Semester: Feb to June)	2020 (%)	2021 (%)
Number of students	40	23
Attendance		
Always present	9 (23%)	14 (62%)
Absent once or twice	25 (63%)	5 (23%)
Absent 3 to 4 times	6 (15%)	4 (15%)
Students’ attitude		
Always come to class prepared	17 (43%)	8 (33%)
Always come to class on time	9 (23%)	12 (50%)
Listen attentively during class	34 (85%)	15 (67%)
Actively participate during class activities	37 (93%)	17 (75%)
Accomplishes assigned tasks on time	27 (68%)	17 (75%)

**Table 2:** Students’ course satisfaction.

Items	2020	2021	Mean
Knowledge gain	4.00	4.08	4.04
Competencies and attitudinal gains	4.25	4.00	4.13
Education and training	4.00	4.08	4.04
Reading and comprehension	4.25	4.00	4.13
Lesson content is interesting	3.83	4.08	3.96
Teaching style helps in learning	4.00	4.08	4.04
Faculty-student interaction is effective	4.50	4.25	4.38
Class atmosphere conducive to learning	4.17	4.25	4.21
Evaluation is effective	4.00	4.17	4.09
Overall satisfaction	4.08	4.17	4.13 ^1^

^1^ Grand mean.

**Table 3:** Top 20 keywords.

Keyword	Frequency (*n*)	Keyword	Frequency (*n*)
courses	26	global	12
EMI	26	language	11
students	25	important	10
English	22	foreign	9
learn	18	opportunities	9
Taiwan	16	abroad	8
able	14	international	8
countries	14	need	8
become	12	open	8
culture	12	study	8

## Data Availability

Data is not available due to privacy concerns.
